# Our Hospital Article

**Published:** 1859-10

**Authors:** 


*Our Hospital Article.*—We depart from our order this month in presenting an account of Bellevue, by Dr. E. B. Dalton. The account of Ward's Island, which was to have appeared, was delayed in consequence of the want of some statistics. This will probably appear in our next.

